# Planning, implementing, and evaluating Hepatitis C virus elimination via collaborative community-based care cascade: age–period–cohort model for estimating demand from antecedent anti-HCV survey

**DOI:** 10.1007/s12072-023-10605-x

**Published:** 2023-11-21

**Authors:** Ting-Yu Lin, Hsiao-Hsuan Jen, Tsung-Hui Hu, Yu-Chin Yao, Tony Hsiu-Hsi Chen, Amy Ming-Fang Yen, Yen-Po Yeh

**Affiliations:** 1 Graduate Institute of Epidemiology and Preventive Medicine, College of Public Health, National Taiwan University, Taipei, Taiwan; 2 New Doctors, Ltd., Taipei, Taiwan; 3 Division of Hepato-Gastroenterology, Department of Internal Medicine, Kaohsiung Chang Gung Memorial Hospital and Chang Gung University College of Medicine, Kaohsiung, Taiwan; 4 Changhua County Public Health Bureau, No.162, Sec. 2, Jhongshan Rd, Changhua City, Changhua County 500 Taiwan; 5 School of Oral Hygiene, College of Oral Medicine, Taipei Medical University, No. 250, Wu-Hsin Street, Taipei, 110 Taiwan; 6 School of Medicine, College of Medicine, National Sun Yat-sen University, Kaohsiung,, 804, Taiwan

**Keywords:** Hepatitis C elimination, HCV care cascade, Community-based screening program, Age–period–cohort model

## Abstract

**Background:**

Estimating the demand for HCV care cascade plays an important role in planning, monitoring, and assessing the performance of introducing a new community-based hepatitis C virus (HCV) elimination program but such an analytic and systematic approach has been barley addressed.

**Methods:**

A new collaborative care program for HCV elimination in the Changhua Community of Taiwan has been offered to a total of 895,353 residents since 2018. To grasp the variation of demand for HCV care cascade across demographic and geographic features in the planning stage, we applied the age–period–cohort spatial model to the antecedent anti-HCV survey enrolling 123,617 participants aged 30 years or older between 2005 and 2018. Based on this precise denominator, we then employed a “before-and-after” study design to routinely evaluate whether the WHO criteria of 90% RNA positive diagnosis and 80% successful treatments could be reached.

**Results:**

The overall demand for HCV care cascade was 4.28% (HCV infection) of the underlying population but a declining trend was noted. The early cohort had a higher demand, whereas the demand of the young cohort decreased with each passing year. The demand also differed by township. The demand, allowing for these variations, for antiviral treatment was 22,362, yielding the WHO target of 12,880 for achieving HCV elimination. With 11,844 successful treatments, the effectiveness of elimination has already reached 92% (11,844/12,880) by the end of 2022.

**Conclusions:**

The demand for HCV care cascade allows health care decision-makers to timely and properly assess the performance of a novel community-based collaborative care program in achieving HCV elimination.

**Supplementary Information:**

The online version contains supplementary material available at 10.1007/s12072-023-10605-x.

## Introduction

Hepatocellular carcinoma (HCC) is still one of the world’s leading causes of death [1, 2]. Its occurrence is mostly associated with two well-known risk factors, hepatitis B (HBV) and C virus (HCV) infections, particularly in the southeastern Asia [2–6]. It is worth noting that, as compared to HBV infection, HCV infection typically exhibits three unique patterns.

Most HCV infection sufferers contracted the disease by a specific method of transmission, such as reusing needles when medical resources were scarce, notably prior to the introduction of disposable needles and during the era of blood donor screening [7]. The impact of such a chronic infection is thought to differ in the light of age groups that correspond to each birth cohort, and the rate of HCV infection rises with earlier birth cohorts. The second characteristic is active viremia of HCV replication in multiple geographic areas and time periods, which may result in cluster infection of HCV for susceptible persons exposed to and infected by active HCV-infected individuals.

According to epidemiological statistics, the third characteristic is connected to the aging carcinogenesis of chronic hepatitis C patients, whose age at beginning is commonly between 50 and 60 years. To forecast the disease burden of HCV infection as it evolves over time and develop an evidence-based intervention for HCV infection, it is critical to understand the effects of age, period, and cohort effects on HCV infection in different geographical regions. Furthermore, the introduction of antiviral therapy, notably direct-acting antiviral drugs (DAA), has shown efficacy in lowering the incidence of HCC. It is critical to estimate the demand for HCV care cascade from anti-HCV test, RNA diagnosis, and treatment with antiviral therapy to meet the demand estimated by the spatiotemporal age–period–cohort analysis mentioned above.

The three aforementioned reasons, along with the estimated amount of antiviral therapy to be provided, explain why an age–period–cohort (APC) analysis is needed. The previous studies have already demonstrated how the APC model is useful for not only examining the effects of age, period, and cohort on the disease burden of HCV incidence [8–10] and mortality [10] but also elucidating respective contributions to the evolution of HCV epidemic in high burden area [11]. All these studies have also revealed the usefulness of the APC model in identifying subpopulations at risk by gender, age groups, birth cohorts, and geographic areas. All these previous studies provide a good rationale for utilizing the APC model to project the demand for HCV cascade care and the necessity of supplying antiviral therapy using estimates learned from empirical data on anti-HCV massive survey. Most importantly, estimating the demand with the APC model plays a crucial role in determining whether the goal of HCV removal through successful treatment can be achieved through routinely monitoring the performance of HCV elimination following the deployment of community-based HCV cascade care [12, 13].

Since 2005, community-based anti-HCV screening has been included into one component of community-based integrated screening in Changhua, Taiwan's middle metropolis [2]. Applying the age–period–cohort (APC) model to such empirical data enables health decision-makers to estimate the demand for HCV care cascade and evaluate whether the successful HCV elimination can be achieved according to the WHO criteria of 90% RNA positive diagnosis and 80% anti-viral treatment [14].

The aims of this study are therefore to first estimate the demand for HCV care, making allowance for demographic and spatial variation, with the application of the APC model to data on anti-HCV test survey in the planning stage of a new community-based collaborative care for HCV elimination program. We then show how to apply the APC-derived demand for monitoring and assessing the performance of this new community-based HCV elimination program in the light of the WHO criteria.

## Materials and methods

**Fig. 1:**
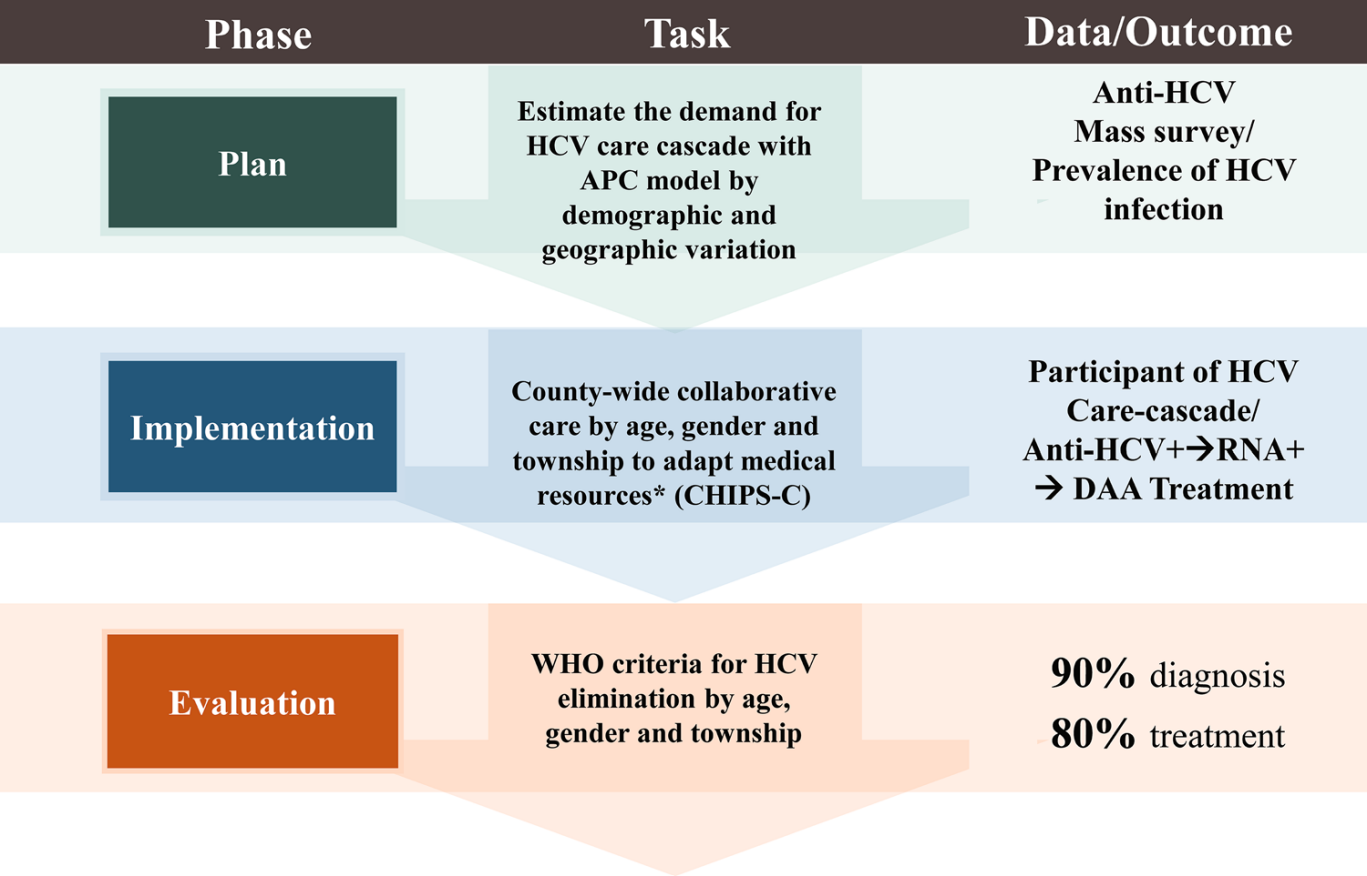
Framework of preparation, implementation, and evaluation of new community-based collaborative HCV care cascade.*Thes include out-reach service, mobile clinical care, and prompt referral system to enhance accessibility

Figure 1 shows the process and the framework of planning, conducting, and evaluating the proposed new community-based collaborative HCV care cascade. In the planning stage, it begins with the estimation of demand for HCV care cascade with the APC model indicated by age-, gender-, and township-specific prevalence rate of HCV infection based on antecedent anti-HCV mass survey test. After then, a county-wide community-based collaborative care program has been organized for implementing HCV elimination for allocating the optimal personnel (such as out-reach service, mobile clinical care, and fast referral system) to meet the demand derived from the APC model. In evaluation phase, we used the WHO criteria of 72% successful treatment rate given 80% viremia to evaluate the performance of HCV elimination by the APC-derived demand, taking demographic and geographic variations into account.

### Changhua community-based anti-HCV test

A community-based anti-HCV test was undertaken by the Health Bureau of Changhua County in Taiwan, which is located in the central island and has a population of roughly 1.27 million people living in 26 administrative townships scattered across a total area of 1074 km^2^. Between 2005 and 2018, 123,617 residents underwent an anti-HCV test out of 124,562 participants in the Changhua Community-based Integrated Screening (CHCIS) program, an integrated community-based service screening program for residents aged 30 and above. The specifics of the CHCIS program can be read elsewhere [2]. It is quite similar in design and implementation to the previously suggested Keelung community-based integrated screening (KCIS) [15]. In brief, the CHCIS program has provided a series of preventive services, with a focus on both neoplastic disease (liver cancer, breast cancer, cervical cancer, oral cancer, colorectal cancer, and gastric cancer) and non-neoplastic disease (hepatitis B/C and alpha-fetoprotein, cirrhosis, tuberculosis, hypertension, hyperlipidemia, hyperglycemia, and depression).

Data from this community-based anti-HCV test were used to estimate the demand for HCV care cascade to achieve eradication based on participants having a positive anti-HCV test result. Participants in the CHCIS program completed questionnaires to provide demographic information, lifestyle habits, family history, and other relevant information, which were eventually included into the CHCIS database.

### Age–period–cohort (APC) model for estimating demand for HCV care cascade

Because HCV infection was once contagious from childhood, estimating the prevalence of such a persistent infection requires knowledge of the year of birth and calendar year to reflect the change in epidemiological profiles concurrent with the evolution of health, medical care, and health hygiene for different age groups while enrolled in the CHCIS program. The data were separated into eleven 5-year age bands ranging from 30–34 to 75–79 and >  = 80, three time periods (2005–2009, 2010–2014, and 2015–2018), and sixteen birth cohorts spanning the years 1912–1916 to 1987–1991. The age–period–cohort (APC) design is used to describe epidemiological profiles of HCV infection prevalence by birth cohort and calendar year. Following that, the Bayesian APC Poisson regression model forecasted the demand for cascade care for HCV treatment by township.

### Changhua-integrated program to stop Hepatitis C infection (CHIPS-C)

In the light of the demand derived from the APC model, the Changhua local government has launched a collaborative care program since 2018 for eliminating HCV with DAA anti-viral therapy through various levels of institutions and primary care settings, targeting various kinds of risk groups, including the PWID group, dialysis patients, chronic kidney disease (CKD), and diabetes mellitus (DM). A group of inter-disciplinary professionals, including gastroenterologists, family physicians, internal medicine physicians, and others from medical institutions, primary care clinics, and health care center carried out the cascade of care from the anti-HCV test, RNA diagnosis, and anti-viral treatment. This collaborative and inter-disciplinary approach has been named the CHIPS-C program. It has been expanded from the Changhua community-based integrated screening program (CHCIS) mentioned earlier to embrace all portals of recruiting participants undergoing anti-HCV test including the health check-up as usual from CHCIS, patients with diabetes mellitus from shared care, patients with CKD from managed care in the health care center, and their equivalents from the vicinity of GI care. However, not all patients who visited GI care have undergone the uptake of anti-HCV test. Only patients with abnormal ALT received anti-HCV test. Doing so was to prioritize the identification of subpopulations at risk for HCV infection from the GI care with efficiency. Unfortunately, the detailed information on the abnormality of ALT for each individual is not available and only the total number of clients (*n* = 662,754) during the study period is known, the proportion of such a kind of those patients visiting GI care was approximated as 40% based on the previous study [16]. Accordingly, the total number of 265,102 was approximated as the denominator of screening rate for undergoing anti-HCV test for the portal of GI care.

It also should be noted that although the PWID group and dialysis patients have been included in our new community-based collaborative care as mentioned above and had high successful treatment rate for HCV elimination that have been already described in full elsewhere [17, 18]. However, these groups were excluded from the current study because these high-risk subpopulations were very unlikely to participate in the community-based integrated screening where anti-HCV community-based surveys were conducted for estimating the demand using the APC model. The demand estimated by the APC model would not include both high risk subpopulations. Similarly, these high-risk subjects would not be recruited through the portals of recruiting participants undergoing anti-HCV test as mentioned above.

Using the demand estimated with the APC model as the denominator, we assessed the completion rate of HCV eradication given the anticipated HCV target of HCV elimination on the basis of 80% RNA positive diagnosis and 90% compliance with treatment.

### Laboratory test of HCV

Anti-HCV antibodies were discovered using chemiluminescent microparticle immunoassay (CMIA) kits (ABBOTT) in 2007, immunoassay kits (Roche) in 2013, and enzyme-linked immunosorbent assay (ELISA) kits (NANBASE C-96 3.0) in 2005–2006, 2008–2012, and after 2014.

### Statistical analysis

The 5-year moving average was used to stratify the time-trend analysis of prevalence into three age groups by gender. As the prevalence of HCV infection depends on age and period, which yields the mixture effect of birth-cohort, we applied the age–period–cohort (APC) model to consider these three effects as functions simultaneously. In our study, data were divided into eleven 5-year age bands (30–34, 35–39, …,75–79, >  = 80), three time periods (2005–2009, 2010–2014, 2015–2018), and 16 birth cohorts (1912–1916, 1917–1921, …,1982–1986, 1987–1991).

The Bayesian APC Poisson regression model with the directed acyclic graphic (DAG) diagram was proposed to estimate the age–period–cohort effect on the HCV infection, as shown in sFigure 1. The model regresses the counts of HCV infection and the corresponding person years on the three main independent variables, including age, period, and cohort, which account for the heterogeneity across the townships, in order to train the intercept and the second order autoregressive function for the regression coefficients of age(${\alpha }_{i,a}$), period(${\beta }_{i,p}$) and cohort(${\gamma }_{i,c}$) effects, where the first two terms for each effect follow non-informative priors with normal distributions $[\mathrm{N}(0,\frac{1{0}^{6}}{{\tau }_{\alpha }})$, $\mathrm{N}(0,\frac{1{0}^{6}}{{\tau }_{\beta }})$, $\mathrm{N}(0,\frac{1{0}^{6}}{{\tau }_{\gamma }})]$. Accordingly, we projected the HCV infection in the following period by sampling from the posterior distribution for each parameter.

The Bayesian–Markov Chain Monte Carlo with Gibbs Sampling algorithm was developed to construct the posterior distribution of the parameters. To have a stationary distribution, we first had 5,000 burn-in samples and then another 20,000 samples to approximate the target distribution. In addition, to avoid the correlation for each sample, only 1 of every 10 samples was selected in the simulation. After obtaining the posterior distribution, we can have the 95% credible interval of all the estimates.

## Results

### Demand derived from prevalence of HCV infection

sTable 1 shows the prevalence of HCV infection in the Changhua community by sex and age, based on data obtained from community-based anti-HCV tests between 2005 and 2018. Overall rate was 4.28% (5288/123,617). The corresponding figures for males and females were 4.27% (1882/44,055) and 4.28% (3406/79,562), respectively. With increasing age, the overall prevalence increased from 1.98 to 9.10%. Males had a higher incidence than females under the age of 50, whereas the opposite was true for those 50 and older.

**Fig. 2:**
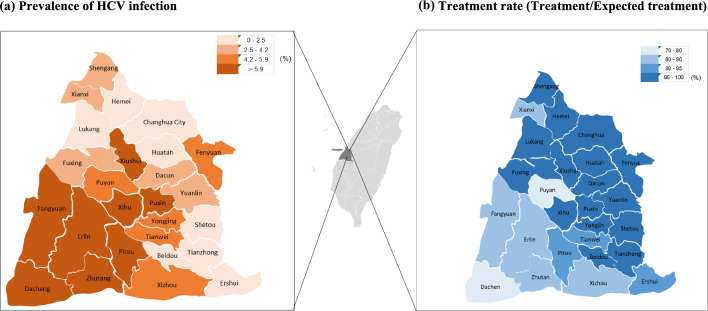
Prevalence of HCV infection and treatment of HCV infection among 26 townships in Changhua County, Taiwan, 2005–2018

**Fig. 3:**
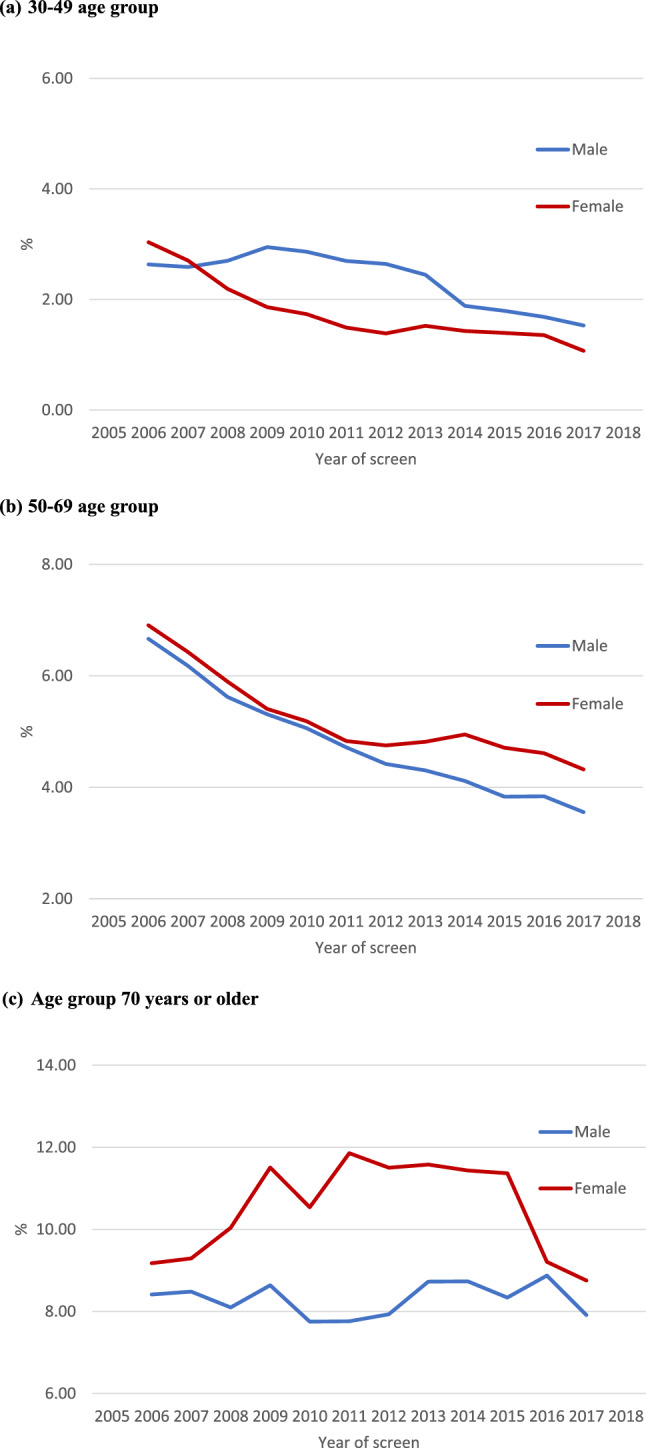
Prevalence of HCV infection by gender among 10-year age groups

Geographic differences in the prevalence of HCV infection between 2005 and 2018 are shown in Fig. 2. Greater prevalence was noted in the southwestern Changhua (coastal areas), but lesser prevalence was observed in the northeastern Changhua. The gender distribution was similar, however the number of townships with a prevalence greater than 5.9% was higher in females than males. Figure 3 shows three age-specific groups of time trends for HCV infection prevalence from 2005 to 2018. While the middle-age group (50–69) experienced significant declines, the oldest age group (> = 70) experienced a slightly increasing trend. Furthermore, gender inequalities by age group were discovered. Males aged 30–49 had a higher prevalence, whereas females aged 50 or older had a higher prevalence.

### Prevalence of HCV infection by birth cohort

**Fig. 4:**
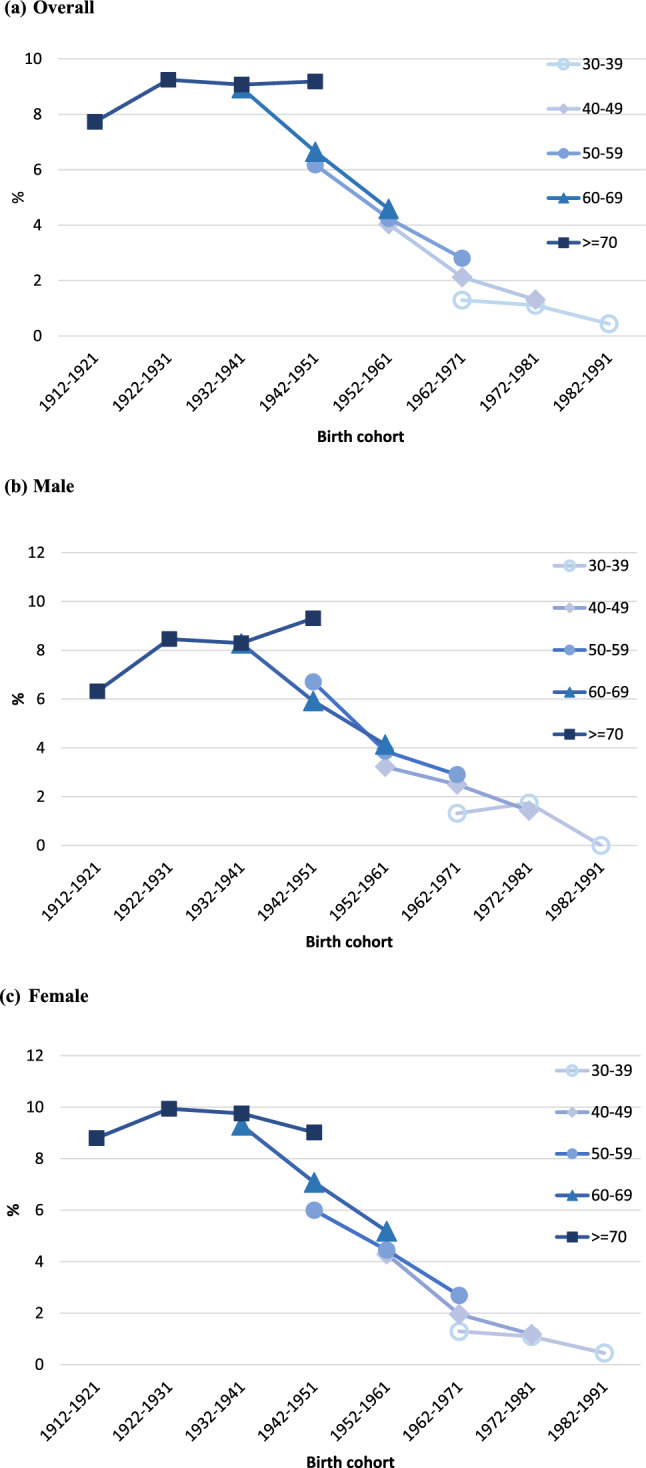
Age–cohort effect on prevalence of HCV infection

Figure 4 shows the age-cohort effect on the prevalence of HCV infection. The frequency increased by cohort for people over 70 years and born between 1912 and 1951, and was more noticeable in males than females. In the younger group, however, the prevalence decreased with the year of birth. The incidence of HCV infection increased in the birth cohort before 1941 but decreased in the birth cohort after 1941. The cohort effect on the prevalence of HCV infection differed significantly when four risk groups were stratified (see sFigure 2).

### Age–period–cohort (APC) models of HCV infection

The findings of APC models in sTable 2 suggest that males have experienced a greater decline in HCV prevalence than females, especially in the middle-aged group (50–69). In comparison to period 1 (2005–2009), HCV prevalence among males in the middle-aged group (50–69) decreased by 21% reduction (95% confidence interval (95% CI): 4–36%) during period 2 (2010–2014) and by 35% (95% CI: 7–57%) during period 3 (2015–2018). The same pattern was observed for the age group in males and the middle- and old-age groups in females, even if the period effects in other age groups and females were not statistically significant.

Based on the APC model, we assessed the prevalence of HCV infection by different genders and county (sFigure 3 and sTable 3) during Period 3 (2015–2018) and also projected the total number of HCV infections in the Changhua population for the next 5-year period of 2019–2023 (Table 1 and sTable 4). During period 3 (2015–2018), there was a minor discrepancy between the observed and the estimated prevalence among both genders. Furthermore, among CHCIS program participants, the estimated number of HCV infection was slightly higher than the observed number in males (569 vs. 542 for estimated vs. observed), but lower in females (473 vs. 483 for estimated vs. observed). Changhua could have 22,362 HCV-infected residents in the next 5 years (2019–2023). The anticipated number of HCV infections was highest in the middle age group (4558, 10,890, and 6914 for the young-, middle-, and older-age groups, respectively), and in females (11,576 vs. 10,786 for female vs. male). In compared to females, the estimated number of males with HCV infection in the young-age group exceeds 450. (2054 vs. 2504 for female vs. male); in the middle-age group, this figure is slightly higher in female (5492 vs. 5398 for female vs. male); in older-age group, there was larger gender difference, with female outnumbering male by 1147 (4030 vs. 2883 for female vs. male). The detailed figures of geographic distribution are listed in sTable 4.

**Table 1:** Estimation of the number of HCV elimination by age group based on APC model in the following 5 years (2019–2023)

Age group	Population	Projected HCV infection		
		Total cases	Male	Female
30–34	97,240	642.3	380.1	262.2
35–39	108,493	1028.7	580.3	448.4
40–44	94,713	1251.0	676.7	574.3
45–49	89,199	1636.3	867.4	768.9
50–54	94,186	2284.1	1208.5	1075.6
55–59	93,891	2881.6	1482.2	1399.4
60–64	80,984	2991.9	1444.4	1547.6
65–69	63,150	2732.5	1262.7	1469.8
70–74	38,225	1884.9	857.9	1027.1
75–79	37,445	2007.2	885.4	1121.8
80 +	50,679	3021.6	1140.1	1881.5
Overall	848,205	22,362.1	10,785.5	11,576.6

Based on the estimated 22,362 HCV positives in Table 1, the anticipated number of HCV viremia infections with APC analysis, after accounting for spatio-temporal variation, was 17,890 with an 80% RNA positivity. Our community-based HCV elimination program would expect 12,880 effective treatments out of 17,890 in order to meet the WHO criteria of 90% diagnosis and 80% treatment [12].

### HCV elimination rate through HCV cascade care of the CHIPS-C

Table 2 shows the results of cascade care in our CHIPS-C program for HCV elimination using both a community-based and an institution-based strategy. Those in the former were largely recruited through shared care for people with diabetes, managed care for persons with chronic kidney disease (CKD), and community-based health check-ups; participants in the latter were nearly equivalent but received cascade care in the hospital for GI care. Table 2 shows the detailed empirical results of the HCV care cascade. Recall that the denominator for the GI care is approximated by the total number of patients during the study multiplied by the proportion of abnormal ALT from the previous study as mentioned in the method section. Of 421,268 participants who had an anti-HCV test, there were 23,871 anti-HCV positives (5.7%). There were 12,930 RNA positives (79.8%) out of 16,202 who had a RNA test, and 11,844 antiviral treatments (91.6%) given to those who were RNA positives. Those who obtained community-based HCV care cascade and successful treatment were slightly fewer than those who received GI care in hospitals (5034 versus 6810). For our community-based HCV care cascade, the completion percentage of effective treatment given RNA positive was 91.6% (11,844/12,930).

**Table 2:** Results of HCV care cascade in Changhua, Taiwan 2018–2022

Indicators		CHIPS-C program			Total
	Early CKD	GI care	DM shared care	Health check-up	
HCV anti-body tested	25,168/34,444 (73.1%)	212,999/265,102^a^ (80.3%)	58,220/68,821 (84.6%)	124,881/129,334 (96.6%)	421,268/497,701 (84.6%)
HCV anti-body positive	1733/25,168 (6.9%)	14,202/212,999 (6.7%)	3483/58,220 (6.0%)	4453/124,881 (3.6%)	23,871/421,268 (5.7%)
HCV RNA tested	1352/1733 (78.0%%)	7999/14,202 (56.3%)	2883/3483 (82.8%)	3968/4453 (89.1%)	16,202/23,871 (67.9%)
HCV RNA positive	867/1352 (64.1%)	7341/7999 (91.8%)	1796/2883 (62.3%)	2926/3968 (73.7%)	12,930/16,202 (79.8%)
Treatment	733/867 (84.5%)	6810/7341 (92.8%)	1615/1796 (89.9%)	2686/2926 (91.8%)	11,844/12,930 (91.6%)

^a^This number is approximated by using the total number of patients seeking for the GI care (*n* = 662,754) × the abnormal rate of ALT (0.4) obtained from the previous study [16] as mentioned in the method section.

Based on the 12,880 projected HCV elimination targets as predicted above, the CHIPS-C program has already completed 92% (11,844/12,880) of HCV removal, ranging from 70 to 100% depending on townships, by the end of 2022.

## Discussion

In a high-prevalence chronic HCV infection area such as Taiwan, it is imperative to assess if and to what extent a community-based HCV care cascade, if provided at the population level, can achieve the target of HCV elimination in light of WHO criteria. However, it is important to estimate the demand for HCV care cascade given heterogeneous socio-demographic and geographic HCV infections before. The current study used a novel design with the APC model to estimate 22,362 candidates for being diagnosed as RNA positive and requiring antivirals based on data from a community-based anti-HCV test between 2005 and 2018. The community of Changhua in Taiwan has approximately 800,000–900,000 residents aged 30 years or older. Of these, 12,880 were further set up as the WHO target for HCV elimination. The projected target was routinely used to monitor the performance of a new community-based care cascade that has been conducted since 2018. Given the 11,844 effective treatments observed from those attending community-based HCV care cascade, a high performance of 92% HCV elimination has been accomplished by the end of 2022 in comparison to the WHO target.

### Age–period–cohort effect on HCV prevalence

The estimated overall prevalence of HCV infection in Changhua during the period of 2005–2018 was 4.28%, which was lower than the earlier survey from a community-based screening program targeting high-risk populations in seven townships of Taiwan in 1991–1992 [19]. However, because HCV chronic infection is affected by environmental hygiene and medical advances in chronological order, resulting in the heterogeneity of HCV determined by age, period, and cohort in different geographic areas, these previous cross-sectional surveys on anti-HCV tests may not provide an unbiased estimate HCV chronic infection in the underlying population, and the APC design and analysis for estimating the demand for HCV care cascade are therefore required.

In our analysis, the declining trend of HCV infection was also seen, especially in the male middle-age group (50–69), where there was a 21% reduction during Period 2 (2010–2014) and a 35% reduction during Period 3 (2015–2018) compared to Period 1 (2005–2009). We also discovered that, whereas the prevalence decreased with age for the younger cohort, it increased for people over the age of 70 born between 1912 and 1951.

Furthermore, the prevalence of HCV infection increased for the birth cohort prior to 1941, but then fell. Such APC findings served as the rationale and the basis for estimating the demand for HCV care cascade and setting up the target of achieving HCV elimination in the light of the WHO criteria, in order to make it a rule to monitor and evaluate the performance of our population-based HCV care cascade since its implementation.

### HCV care cascade elimination worldwide

To achieve the WHO’s goal of eradicating HCV by 2030, a 90% screening (diagnostic) rate and an 80% treatment rate were established. As a result, many HCV screening elimination programs are being implemented for various target populations, including the general population and high-risk/specific populations (i.e. hospital inpatients, human immunodeficiency virus (HIV)-infected patients, baby-boomers, migrants, PWID, prisoners, pregnant female, and the homeless population etc.). In 2012, the United States Centers for Disease Control and Prevention (CDC) advocated universal screening for the whole birth cohort 1945–1965 (baby-boomers) [20]. Furthermore, in 2018, the Egyptian government initiated a national screening program for the general population over the age of 18 (about 62.5 million individuals) to identify and treat all HCV-infected subjects in order to accomplish HCV elimination [21]. The screening rate was 79.4% from October 1, 2018 to April 30, 2019, resulting in a 4.61% prevalence of HCV infection among untreated people. Furthermore, 91.8% of individuals with HCV RNA positive infections began treatment, and 65.1% completed it by September 30, 2019. It should be noted that while these HCV screening programs had been successfully implemented with feasibility, evaluating whether and to what extent these programs achieved the goal of HCV elimination in the light of the WHO criteria is difficult. This provides a compelling reason to estimate rationale for estimating the demand for HCV care cascade with the APC model before the implementation of a community-based HCV care cascade.

### Collaborative community-based HCV care cascade in Taiwan

To the best of our knowledge, this is the first study to offer multifaceted HCV screening strategies in Changhua, Taiwan under a collaborative community-based care cascade for eliminating HCV with DAA anti-viral therapy through different levels of institutions and primary care settings, targeting various risk groups, including the PWID group, dialysis patients, pre-ESRD, chronic kidney disease. Participants in the three latter groups were enrolled in our community-based integrated screening program (CHCIS) and all GI care institutions in a community-based care cascade. It should be noted that when evaluating the performance of community-based HCV elimination, we excluded the high-risk population, which included dialysis, pre-ESRD, and PWID (HIV-infected and prisoners), because we estimated the demand for HCV care cascade using the APC design and analysis based on data from the CHCIS program’s general population. These high-risk populations would not be served by CHCIS or GI care participants.

There are three limitations of this study. First, our current study lays emphasis on HCV elimination  targeting for individuals recruited in diabetes shared care and CKD managed care, as well as those invited to health check-ups and their equivalents from the GI care. We excluded the traditional high-risk group, such as the PWID group and dialysis patients with the reason explained earlier. As a result, our findings cannot be applied to these specific populations with the current methodology. However, their methodology and their results for evaluating the successful anti-viral treatment group have been detailed elsewhere [17, 18].

The second limitation is that, while estimating the demand for HCV care cascade based on age, calendar year, and year of birth by township can reflect geographical variation in prevalent HCV infection, the inference for estimating demand is still limited to the township-specific level rather than the individual level because we lack individual attributes such as socioeconomic status, education, lifestyle factors, and so on. As a result, ongoing research should address how to achieve HCV elimination from the aggregate group level to the sub-group or individual level while incorporating the detailed correlates.

The third is that the denominator for the screening rate for those with abnormal ALT recruited from the GI care (Table 2) is approximated as mentioned in the method section rather than from individual data. This may be validated in the further research by using the sampling-based method. However, we believe this would not seriously affect the results of HCV elimination through our new community-based collaborative care model as the demand of HCV infection required for successful treatment is independently estimated by the application of the APC model.

In conclusion, estimating the demand for HCV care cascade in planning stage plays a crucial role in setting up the target of achieving HCV elimination in light of the WHO criteria. Do so is very helpful for health decision-makers to timely monitor and evaluate the success of community-based HCV care cascade for achieving elimination. Following the application of the APC model in predicting the demand for HCV care cascade allowing for demographic and geographic variations, high performance of a community-based care cascade in HCV eradication through organized collaborative care in one Taiwanese community was demonstrated.

### Supplementary Information

Below is the link to the electronic supplementary material.

Supplementary file1 (DOCX 477 KB)

## Data Availability

The data used during this study are available from the corresponding author upon reasonable request.
