# Supplementary material for: Planning, implementing, and evaluating Hepatitis C virus elimination via collaborative community-based care cascade: age–period–cohort model for estimating demand from antecedent anti-HCV survey
Source: Hepatol Int. 2023 Nov 21;18(2):476–85. doi: 10.1007/s12072-023-10605-x (PMC11014808; doi:10.1007/s12072-023-10605-x)
Supplement: Supplementary file 1 — Supplementary file1 (DOCX 477 KB) [file 12072_2023_10605_MOESM1_ESM.docx]

**Supplementary Materials**

**sFigure 1. The Bayesian directed acyclic graphic (DAG) model for the age, period, and cohort effects on the HCV infection within township level, Changhua**


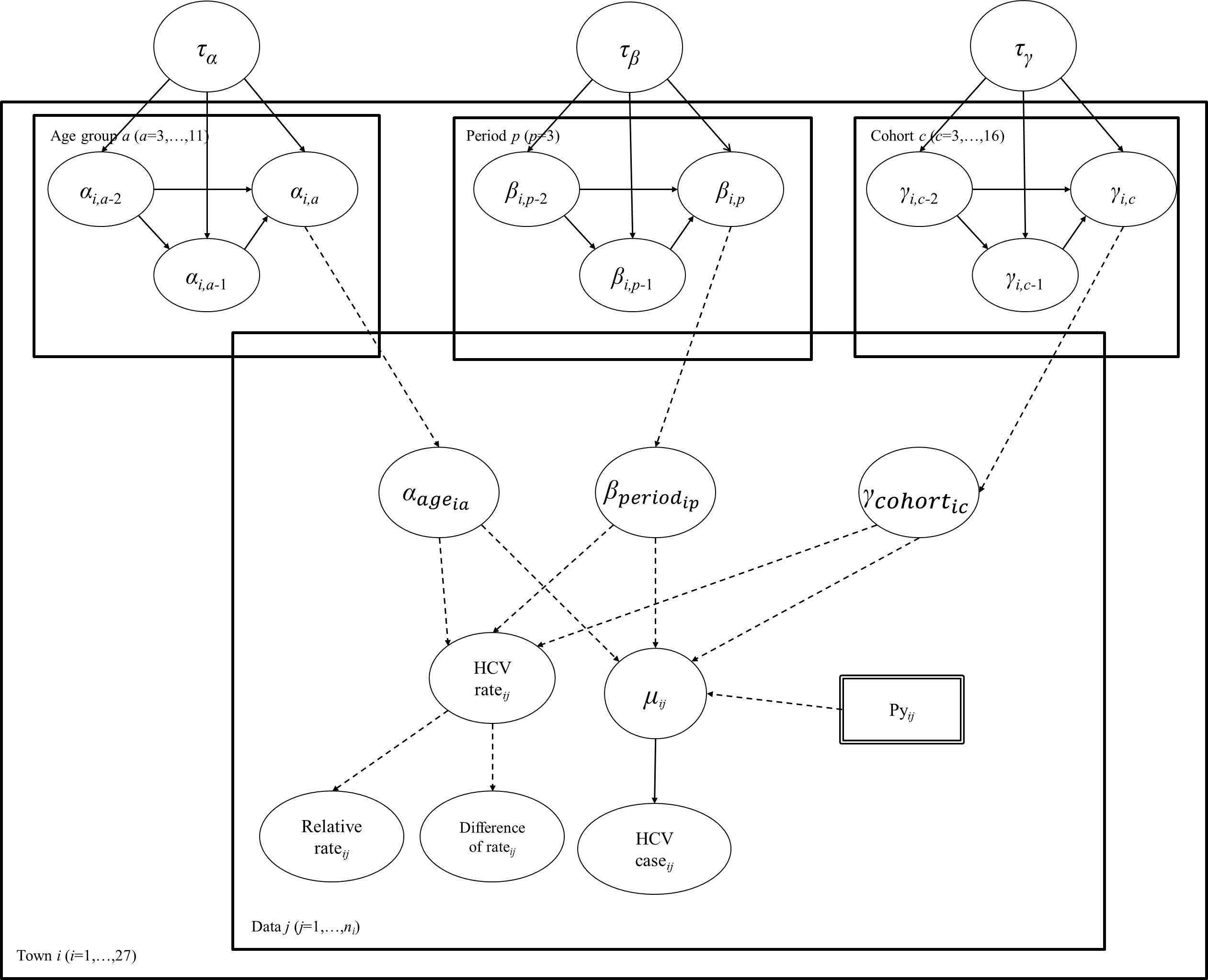


**The Bayesian age-period-cohort (APC) Poisson regression model with the directed acyclic graphic (DAG)**

This Bayesian DAG model regresses the counts of HCV cases (denoted by ${HIV case}_{ij}$) and the corresponding person years (denoted by $PY_{ij}$) on the three main independent variables, including age ($\alpha_{ia}$), period ($\beta_{ip}$) and cohort ($\gamma_{ic}$) as follows,

$HCV case_{ij}\sim Poisson\left( \mu_{ij} \right)$ (1)

$\log\left( \mu_{ij} \right)=\log\left( PY_{ij} \right)+\alpha_{ia}+\beta_{ip}+\gamma_{ic}$ (2)

$$i=1,2,\ldots,27$$

$$a=1,2,\ldots,11$$

$$p=1,2,\ldots,3$$

$$c=1,2,\ldots,16$$

Where $\alpha_{ia}$represent the age effect on the HCV infection with 11 age bands (denoted by a) across 27 townships (denoted by i), $\beta_{ip}$and $\gamma_{ic}$represent the period (denoted by p) and cohort effects (denoted by c), respectively, across 27 townships.

For the specification of the prior distributions, the parameters of first term ($\alpha_{i,1}, \beta_{i,1} and \gamma_{i,1})$and second term ($\alpha_{i,2}, \beta_{i,2} and \gamma_{i,2})$ of age, period and cohort follow non-informative Normal distribution with high variance [Eq. (3) (5) and (7)] and informative normal distribution with mean following second-order autoregression function (eg. $\alpha_{a,j}|\alpha_{a-1,j},\alpha_{a-2,j}\sim N\left( 2\alpha_{a-1,j}-\alpha_{a-2,j},\frac{1}{\tau_{\alpha}} \right)$ for age effect) are specified for the third and the further parameters. [Eq. (4) (6) and (8)]

$\alpha_{i,1}\sim N\left( 0,\frac{10^{6}}{\tau_{\alpha}} \right) , \alpha_{i,2}|\alpha_{i,1}\sim N\left( 0,\frac{10^{6}}{\tau_{\alpha}} \right),$ (3)

$\alpha_{i,a}|\alpha_{i,a-1},\alpha_{i,a-2}\sim N\left( 2\alpha_{i,a-1}-\alpha_{i,a-2},\frac{1}{\tau_{\alpha}} \right), a=3,\ldots,11$ (4)

$\beta_{i,1}\sim N\left( 0,\frac{10^{6}}{\tau_{\alpha}} \right), \beta_{i,2}|\beta_{i,1}\sim N\left( 0,\frac{10^{6}}{\tau_{\alpha}} \right),$ (5)

$\beta_{i,p}|\beta_{i,p-1},\beta_{i,p-2}\sim N\left( 2\beta_{i,p-1}-\beta_{i,p-2},\frac{1}{\tau_{\alpha}} \right), p=3$ (6)

$\gamma_{i,1}\sim N\left( 0,\frac{10^{6}}{\tau_{\gamma}} \right), \gamma_{i,2}|\gamma_{i,1}\sim N\left( 0,\frac{10^{6}}{\tau_{\gamma}} \right),$ (7)

$\gamma_{i,c}|\gamma_{i,c-1},\gamma_{i,c-2}\sim N\left( 2\gamma_{i,c-1}-\gamma_{i,c-2},\frac{1}{\tau_{c}} \right), c=3,...,16$ (8)

The DAG model (sFigure 1) shows the parents-children relationship for the relevant variables and regression coefficients, for example, the observed counts of HCV cases (${HCV case}_{ij}$) following Poisson distribution with mean determined by person years and incidence rate, and also a series of functions on age, period, and cohort effects, each of which is captured by the two previous terms, taking age as an example $(\alpha_{a-1,j}$, $\alpha_{a-2,j} )$, and precision ($\tau_{\alpha})$.

**sFigure 2. Age-cohort effect on prevalence of HCV infection by level**

1. **Prevalence < 2.5%**
2. **Prevalence 2.5-4.2%**
3. **Prevalence 4.2-5.9%**
4. **Prevalence >5.9%**

**sFigure 3. Geographic distribution of the estimated number of HCV infection with APC model by gender between 2015-2018**

1. **Male (B) Female**


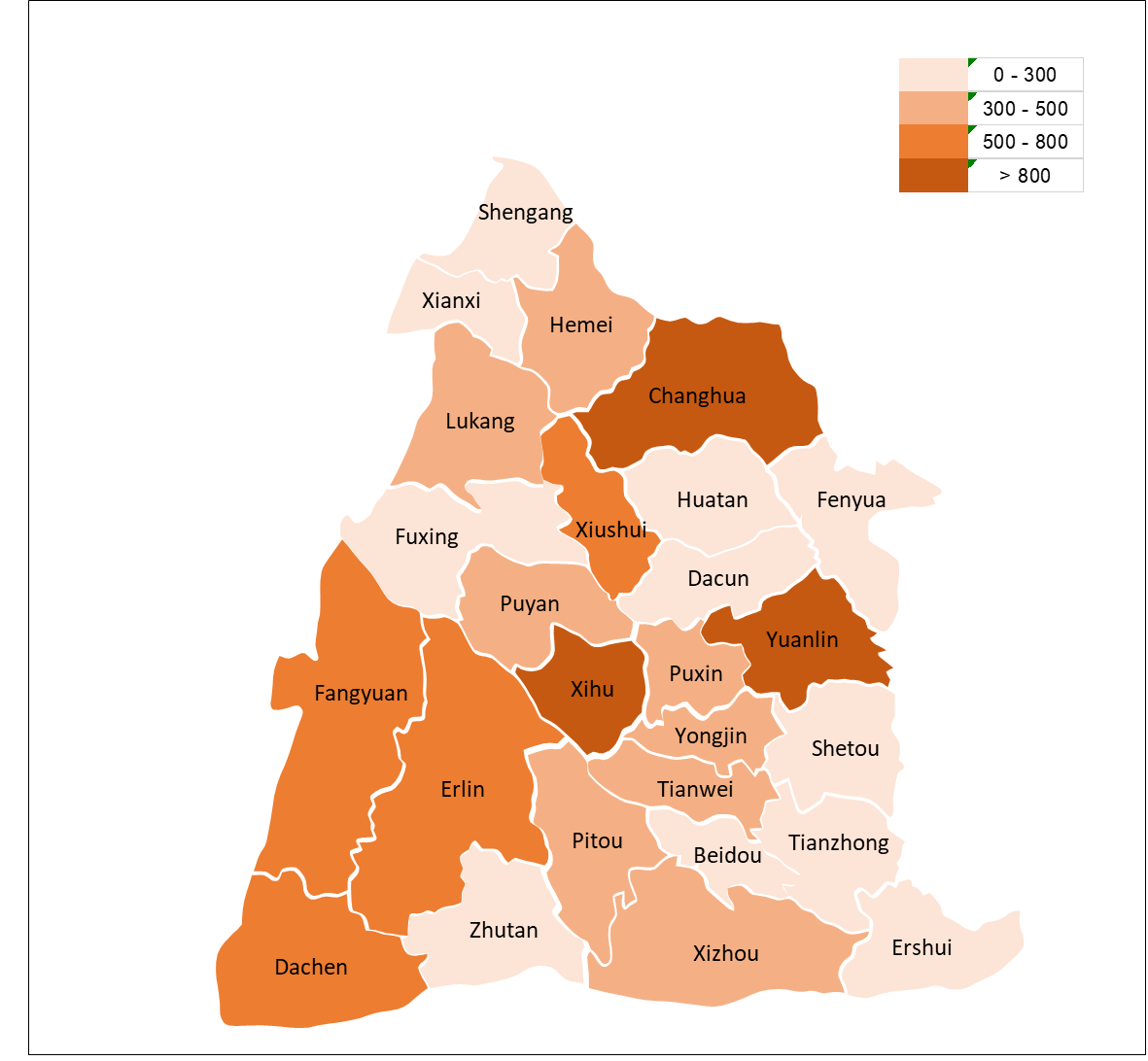

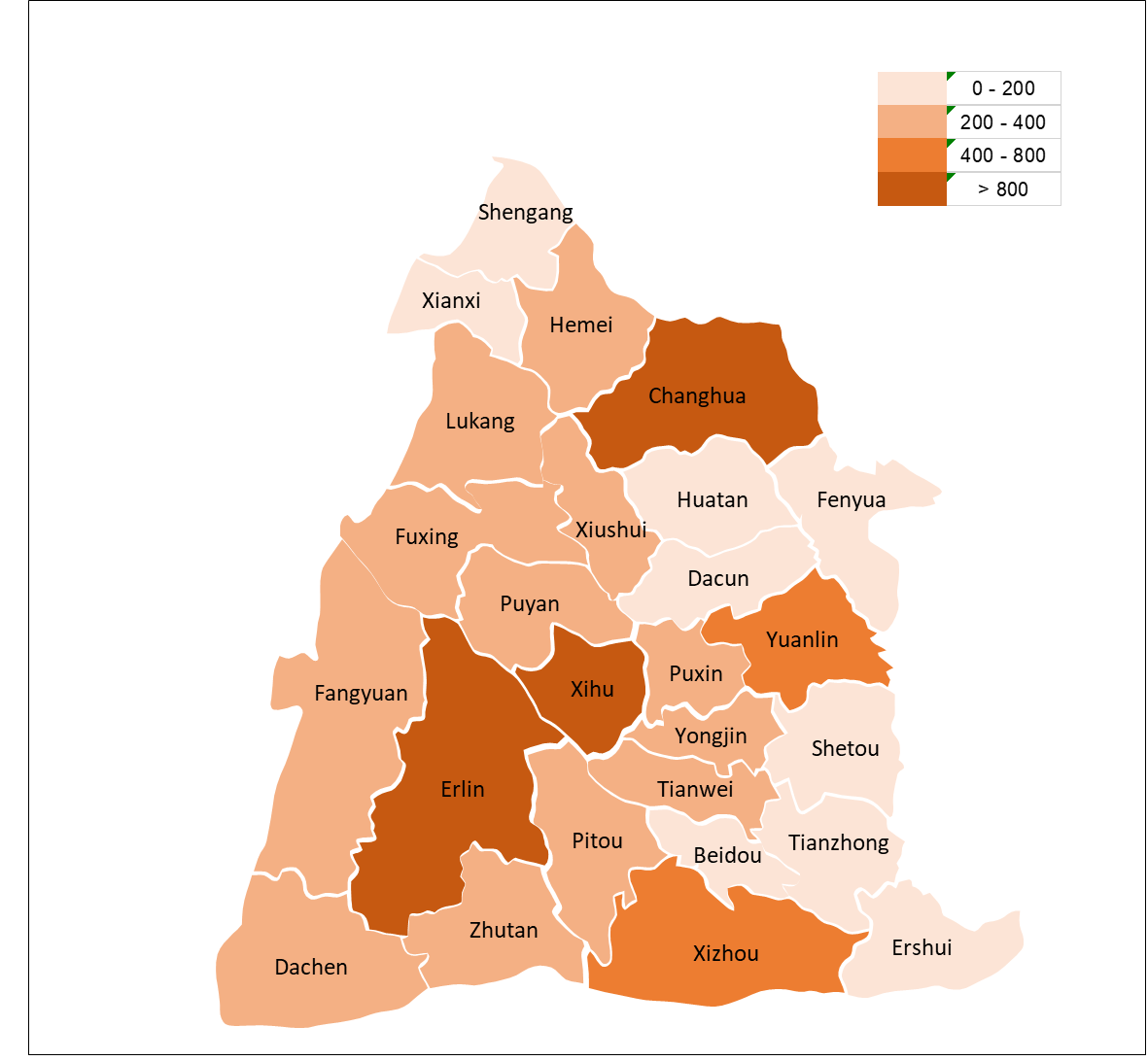


**sTable 1. Prevalence of HCV infection by age and gender in Changhua community**

| **Age group** | **Male** | | | **Female** | | | **Overall** | | |
| --- | --- | --- | --- | --- | --- | --- | --- | --- | --- |
|  | **Prevalent case** | **Screenee** | **Prevalence**  **(%)** | **Prevalent case** | **Screenee** | **Prevalence**  **(%)** | **Prevalent case** | **Screenee** | **Prevalence**  **(%)** |
| **30-49** | 277 | 12752 | 2.17 | 626 | 32762 | 1.91 | 903 | 45514 | 1.98 |
| **50-69** | 1264 | 27234 | 4.64 | 2338 | 42266 | 5.53 | 3602 | 69500 | 5.18 |
| **>=70** | 341 | 4069 | 8.38 | 442 | 4534 | 9.75 | 783 | 8603 | 9.10 |
| **Total** | 1882 | 44055 | 4.27 | 3406 | 79562 | 4.28 | 5288 | 123617 | 4.28 |

**sTable 2. Multivariable Analysis by Gender on Prevalence of HCV**

|  | | Male | | | Female | | |
| --- | --- | --- | --- | --- | --- | --- | --- |
|  |  | aRR | 95% CI | | aRR | 95% CI | |
| Age group |  |  |  |  |  |  |  |
| 30-49 | 2015-2018 | 0.74 | (0.34 | 1.40) | 1.08 | (0.60 | 1.81) |
|  | 2010-2014 | 0.85 | (0.59 | 1.17) | 1.02 | (0.77 | 1.33) |
|  | 2005-2009 | 1.00 |  |  | 1.00 |  |  |
| 50-69 | 2015-2018 | 0.65 | (0.43 | 0.93) | 0.82 | (0.60 | 1.11) |
|  | 2010-2014 | 0.79 | (0.64 | 0.96) | 0.89 | (0.75 | 1.05) |
|  | 2005-2009 | 1.00 |  |  | 1.00 |  |  |
| >=70 | 2015-2018 | 0.69 | (0.43 | 1.06) | 0.84 | (0.54 | 1.27) |
|  | 2010-2014 | 0.85 | (0.65 | 1.08) | 0.91 | (0.71 | 1.14) |
|  | 2005-2009 | 1.00 |  |  | 1.00 |  |  |

**sTable 3. Geographic distribution of number of HCV infection cases between 2015-2018**

| **County** | **Male** | | **Female** | |
| --- | --- | --- | --- | --- |
|  | **Observed** | **Estimated** | **Observed** | **Estimated** |
| Changhua City | 38 | 47.3 | 37 | 35.3 |
| Lukang | 20 | 19.6 | 23 | 18.9 |
| Hemei | 16 | 16.6 | 23 | 16.3 |
| Beidou | 8 | 9.5 | 9 | 8.2 |
| Yuanlin | 35 | 32.2 | 26 | 28.2 |
| Xihu | 52 | 56.1 | 33 | 40.3 |
| Tianzhong | 14 | 13.6 | 9 | 9.2 |
| Erlin | 17 | 18.6 | 22 | 19.4 |
| Xianxi | 12 | 9.5 | 8 | 7.1 |
| Shengang | 12 | 11.4 | 8 | 10.9 |
| Fuxing | 16 | 15.8 | 9 | 10.7 |
| Xiushui | 23 | 31.0 | 12 | 17.7 |
| Huatan | 9 | 10.1 | 8 | 10.0 |
| Fenyuan | 11 | 12.8 | 16 | 16.6 |
| Dacun | 12 | 16.6 | 13 | 12.5 |
| Puyan | 22 | 25.4 | 30 | 22.6 |
| Puxin | 20 | 21.8 | 18 | 15.9 |
| Yongjing | 25 | 24.4 | 12 | 20.1 |
| Shetou | 16 | 13.4 | 10 | 10.7 |
| Ershui | 9 | 7.2 | 6 | 4.7 |
| Tianwei | 29 | 22.4 | 20 | 17.2 |
| Pitou | 28 | 26.6 | 22 | 21.1 |
| Fangyuan | 32 | 28.8 | 17 | 16.0 |
| Dacheng | 40 | 45.0 | 48 | 43.3 |
| Zhutang | 16 | 20.1 | 15 | 16.4 |
| Xizhou | 10 | 13.2 | 29 | 24.0 |
| **Overall** | 542 | 569.0 | 483 | 473.3 |

**sTable 4. Geographic distribution of estimated number of HCV infection cases between 2019-2023 based on APC model**

1. **Overall**

| **County** | **Population** | **Projected HCV infection** | | |
| --- | --- | --- | --- | --- |
|  |  | **Total cases** | **Male** | **Female** |
| Changhua City | 151887 | 2299.4 | 987.4 | 1312.0 |
| Lukang | 55633 | 862.1 | 430.7 | 431.4 |
| Hemei | 59160 | 737.7 | 340.8 | 396.9 |
| Beidou | 21890 | 337.6 | 134.8 | 202.8 |
| Yuanlin | 80554 | 1730.7 | 936.9 | 793.8 |
| Xihu | 35269 | 2204.2 | 1127.8 | 1076.4 |
| Tianzhong | 28487 | 467.4 | 234.0 | 233.4 |
| Erlin | 34762 | 1508.0 | 586.4 | 921.6 |
| Xianxi | 11081 | 332.6 | 161.3 | 171.3 |
| Shengang | 23659 | 460.2 | 197.1 | 263.1 |
| Fuxing | 31012 | 645.1 | 272.4 | 372.7 |
| Xiushui | 25153 | 971.2 | 545.4 | 425.9 |
| Huatan | 30571 | 433.6 | 148.4 | 285.1 |
| Fenyuan | 16405 | 428.1 | 158.7 | 269.4 |
| Dacun | 24616 | 520.3 | 277.7 | 242.6 |
| Puyan | 22278 | 956.5 | 498.7 | 457.9 |
| Puxin | 23475 | 746.6 | 370.0 | 376.7 |
| Yongjing | 24986 | 822.6 | 442.1 | 380.5 |
| Shetou | 28695 | 491.6 | 242.3 | 249.3 |
| Ershui | 11213 | 243.2 | 149.5 | 93.7 |
| Tianwei | 18306 | 774.3 | 405.1 | 369.3 |
| Pitou | 20689 | 906.2 | 440.3 | 466.0 |
| Fangyuan | 24164 | 1047.3 | 587.7 | 459.6 |
| Dacheng | 12670 | 983.2 | 520.0 | 463.2 |
| Zhutang | 10729 | 625.7 | 289.2 | 336.6 |
| Xizhou | 20861 | 826.7 | 301.1 | 525.6 |
| **Overall** | 848205 | 22362.1 | 10785.5 | 11576.6 |

1. **30-49 years-old**

| **County** | **Population** | **Projected HCV infection** | | |
| --- | --- | --- | --- | --- |
|  |  | **Total cases** | **Male** | **Female** |
| Changhua City | 71314 | 649.2 | 242.9 | 406.4 |
| Lukang | 26996 | 178.0 | 118.9 | 59.2 |
| Hemei | 29297 | 167.2 | 70.0 | 97.1 |
| Beidou | 9964 | 66.8 | 25.6 | 41.2 |
| Yuanlin | 37665 | 443.5 | 289.3 | 154.2 |
| Xihu | 16549 | 410.7 | 245.8 | 164.9 |
| Tianzhong | 12115 | 109.3 | 71.3 | 38.0 |
| Erlin | 14813 | 202.2 | 84.3 | 117.8 |
| Xianxi | 5188 | 66.1 | 41.5 | 24.7 |
| Shengang | 11802 | 128.6 | 51.0 | 77.6 |
| Fuxing | 14400 | 116.6 | 48.3 | 68.4 |
| Xiushui | 11864 | 152.7 | 113.9 | 38.8 |
| Huatan | 14537 | 113.6 | 42.0 | 71.5 |
| Fenyuan | 6939 | 78.3 | 30.7 | 47.6 |
| Dacun | 11574 | 128.3 | 85.6 | 42.7 |
| Puyan | 9829 | 245.8 | 142.3 | 103.5 |
| Puxin | 11099 | 130.6 | 75.0 | 55.6 |
| Yongjing | 11348 | 201.9 | 160.3 | 41.6 |
| Shetou | 12905 | 131.0 | 75.0 | 56.0 |
| Ershui | 4268 | 77.9 | 51.9 | 26.0 |
| Tianwei | 8061 | 172.2 | 106.1 | 66.1 |
| Pitou | 8997 | 73.2 | 42.5 | 30.7 |
| Fangyuan | 10083 | 154.0 | 81.0 | 73.0 |
| Dacheng | 5059 | 123.9 | 89.0 | 34.9 |
| Zhutang | 4199 | 70.5 | 41.8 | 28.8 |
| Xizhou | 8780 | 166.2 | 78.5 | 87.7 |
| **Overall** | 389645 | 4558.2 | 2504.4 | 2053.8 |

1. **50-69 years-old**

| **County** | **Population** | **Projected HCV infection** | | |
| --- | --- | --- | --- | --- |
|  |  | **Total cases** | **Male** | **Female** |
| Changhua City | 60376 | 1302.6 | 586.3 | 716.3 |
| Lukang | 21018 | 418.0 | 217.0 | 201.0 |
| Hemei | 23051 | 378.5 | 147.4 | 231.1 |
| Beidou | 8654 | 183.1 | 83.1 | 100.0 |
| Yuanlin | 32352 | 832.0 | 422.3 | 409.6 |
| Xihu | 13749 | 1138.1 | 546.3 | 591.7 |
| Tianzhong | 11444 | 229.3 | 128.6 | 100.7 |
| Erlin | 13333 | 610.2 | 273.7 | 336.5 |
| Xianxi | 4380 | 141.2 | 83.8 | 57.4 |
| Shengang | 8862 | 218.6 | 104.0 | 114.6 |
| Fuxing | 12028 | 346.2 | 183.4 | 162.9 |
| Xiushui | 9975 | 507.5 | 304.0 | 203.6 |
| Huatan | 12180 | 253.8 | 83.2 | 170.6 |
| Fenyuan | 6560 | 190.5 | 81.4 | 109.1 |
| Dacun | 9873 | 289.5 | 143.5 | 146.0 |
| Puyan | 8387 | 422.5 | 219.8 | 202.7 |
| Puxin | 9215 | 368.7 | 193.2 | 175.4 |
| Yongjing | 9915 | 390.9 | 204.1 | 186.8 |
| Shetou | 11542 | 266.8 | 138.4 | 128.4 |
| Ershui | 4531 | 103.4 | 61.7 | 41.7 |
| Tianwei | 7168 | 401.0 | 206.8 | 194.2 |
| Pitou | 8003 | 428.3 | 232.7 | 195.6 |
| Fangyuan | 8831 | 445.4 | 244.4 | 201.1 |
| Dacheng | 4553 | 387.5 | 228.0 | 159.4 |
| Zhutang | 4161 | 288.3 | 139.6 | 148.7 |
| Xizhou | 8070 | 348.4 | 141.0 | 207.4 |
| **Overall** | 332211 | 10890.1 | 5397.7 | 5492.4 |

1. **>=70 years-old**

| **County** | **Population** | **Projected HCV infection** | | |
| --- | --- | --- | --- | --- |
|  |  | **Total cases** | **Male** | **Female** |
| Changhua City | 20197 | 347.6 | 158.2 | 189.3 |
| Lukang | 7619 | 266.0 | 94.8 | 171.2 |
| Hemei | 6812 | 192.0 | 123.4 | 68.6 |
| Beidou | 3272 | 87.7 | 26.1 | 61.6 |
| Yuanlin | 10537 | 455.3 | 225.3 | 230.0 |
| Xihu | 4971 | 655.5 | 335.7 | 319.8 |
| Tianzhong | 4928 | 128.8 | 34.0 | 94.8 |
| Erlin | 6616 | 695.7 | 228.4 | 467.3 |
| Xianxi | 1513 | 125.3 | 36.0 | 89.3 |
| Shengang | 2995 | 113.0 | 42.1 | 70.9 |
| Fuxing | 4584 | 182.3 | 40.8 | 141.5 |
| Xiushui | 3314 | 311.0 | 127.5 | 183.5 |
| Huatan | 3854 | 66.2 | 23.2 | 43.0 |
| Fenyuan | 2906 | 159.3 | 46.6 | 112.7 |
| Dacun | 3169 | 102.5 | 48.6 | 53.9 |
| Puyan | 4062 | 288.2 | 136.5 | 151.7 |
| Puxin | 3161 | 247.3 | 101.7 | 145.6 |
| Yongjing | 3723 | 229.9 | 77.8 | 152.1 |
| Shetou | 4248 | 93.9 | 29.0 | 64.9 |
| Ershui | 2414 | 61.9 | 35.9 | 26.0 |
| Tianwei | 3077 | 201.1 | 92.2 | 108.9 |
| Pitou | 3689 | 404.8 | 165.1 | 239.7 |
| Fangyuan | 5250 | 447.9 | 262.3 | 185.6 |
| Dacheng | 3058 | 471.9 | 203.0 | 268.9 |
| Zhutang | 2369 | 266.9 | 107.8 | 159.1 |
| Xizhou | 4011 | 312.1 | 81.6 | 230.5 |
| **Overall** | 126349 | 6913.8 | 2883.4 | 4030.4 |
